# Sars-Cov-2 in children – insights and conclusions from the mandatory reporting data in Frankfurt am Main, Germany, March–July 2020

**DOI:** 10.3205/dgkh000359

**Published:** 2020-10-09

**Authors:** Ursel Heudorf, Katrin Steul, René Gottschalk

**Affiliations:** 1 Public Health Department of the City of Frankfurt am Main, Germany

**Keywords:** SARS-CoV-2, COVID-19, children, mandatory reporting, public health department, symptoms, transmission routes

## Abstract

**Introduction:** From the beginning of the corona pandemic until August 19, 2020, more than 21,989,366 cases have been reported worldwide – 228,495 in Germany alone, including 12,648 children aged 0–14. In many countries, the proportion of infected children in the total population is comparatively low; in addition, children often have no or milder symptoms and are less likely to transmit the pathogen to adults than the other way round. Based on the registration data in Frankfurt am Main, Germany, the symptoms of children in comparison with adults and the likely routes of transmission are presented below.

**Materials and methods:** The documentation of the mandatory reports includes personal data (name, date of birth, gender, place of residence), disease characteristics (date of report, date of onset of the disease, symptoms), possible contact persons (family, others) and i.a. possible activity or care in children’s community facilities. All reports were viewed, especially with regard to likely transmission routes.

**Results:** From March 1 to July 31, 2020, 1,977 infected people were reported, including 138 children between the ages of 0 and 14 years. Children had fewer and milder symptoms than adults. None of the children experienced severe respiratory symptoms or the need for ventilation. 62% of the children had no symptoms at all (19% adults), 5% of the children were hospitalized (24% adults), and none of the children died (3.8% adults).

After excluding a cluster of 34 children from refugee accommodations and 14 children from a parish, 78% of the remaining 90 children had been infected by an adult within the family, and only 4% were likely to have a reverse transmission route. In 5.5% of cases, transmission in a community facility was likely.

**Discussion:** The results of the registration data from Frankfurt am Main, Germany confirm the results published in other countries: Children are less likely to become infected, and if infected, their symptoms are less severe than in adults, and they are apparently not the main drivers of virus transmission. Therefore, scientific medical associations strongly recommend reopening schools.

## Zusammenfassung

**Einführung:** Seit Beginn der Corona-Pandemie sind bis 19.08.2020 weltweit mehr als 21.989.366 Fälle gemeldet worden – alleine in Deutschland 228.495, darunter 12.648 Kinder im Alter von 0–14 Jahren. In vielen Ländern ist der Anteil der infizierten Kinder in der Gesamtpopulation vergleichsweise gering, darüber hinaus haben Kinder häufig keine oder leichtere Symptome und übertragen den Erreger seltener aus Erwachsene als dies umgekehrt geschieht. Anhand der Meldedaten aus Frankfurt am Main, Deutschland, sollen nachfolgend die Symptome von Kindern im Vergleich mit Erwachsenen sowie die wahrscheinlichen Übertragungswege dargestellt werden.

**Material und Methoden:** Die Dokumentation der Meldungen umfassen persönliche Daten (Name, Geburtsdatum, Geschlecht, Wohnort), Krankheitscharakteristika (Meldedatum, Datum des Erkrankungsbeginns, Symptome), mögliche Kontaktpersonen (Familie, andere) und u.a. eventuelle Tätigkeit oder Betreuung in Kindergemeinschaftseinrichtungen. Alle Meldungen wurden gesichtet, insbesondere auch im Hinblick auf wahrscheinliche Übertragungswege.

**Ergebnisse:** Von 1. März bis 31. Juli 2020 wurden 1.977 infizierte Personen gemeldet, darunter 138 Kinder im Alter von 0 bis 14 Jahren. Kinder hatten weniger und leichtere Symptome als Erwachsene. Schwere Atemwegssymptome oder eine Beatmungspflicht traten bei keinem der Kinder auf. 62% der Kinder hatten keine Symptome (19% Erwachsene), 5% der Kinder wurden in ein Krankenhaus eingewiesen (24% Erwachsene), kein Kind verstarb (3,8% Erwachsene).

Nach Ausschluss von einem Cluster von 34 Kinder aus Flüchtlingsunterkünften und von 14 Kindern aus einer Kirchengemeinde zeigte sich bei den verbliebenen 90 Kindern, dass 78% von ihnen innerhalb der Familie von einem Erwachsenen infiziert worden waren, und nur in 4% war ein umgekehrter Übertragungsweg wahrscheinlich. Bei 5,5% der Fälle war eine Übertragung in einer Kindergemeinschaftseinrichtung wahrscheinlich.

**Diskussion:** Die Ergebnisse der Meldedaten aus Frankfurt am Main, Deutschland bestätigen die aus anderen Ländern publizierten Ergebnisse: Kinder erkranken seltener und weniger schwer als Erwachsene und sind offenbar nicht die Haupt-Überträger des Virus. Vor diesem Hintergrund empfehlen medizinische Fachgesellschaften dringend die Wiederöffnung der Schulen.

## Introduction

Since the beginning of the corona pandemic, 21,989,366 cases infected with SARS-CoV-2 have been reported worldwide as of August, 19, 2020 [1]. In Germany, the number of infected people and those who tested positive is 228,495 (as of August 19, 2020), including 12,648 children aged 0–14 [2].

By now, thousands of scientific publications on COVID-19 have been published, often as advance publications before the expert review process (Medline as of August 19, 2020: SARS CoV 2: 24,194, COVID: 42,021). Without exception, studies show that the proportion of children among the infected population was low in all countries, the symptoms in children were rather mild, many infected children remain asymptomatic, and deaths are extremely rare [3], [4], [5], [6], [7], [8], [9], [10], [11]. This was shown by a systematic review published on March 20, 2020, including children from China, Italy and the USA [9]. Even in Italy, which suffered from high mortality among older adults, children were much less severely affected and recovered completely [8]. A systematic review of hospitalized children in different countries as well as initial data from Germany confirms the data from abroad [12], [13].

Possible explanations for the milder courses in children include a not yet fully developed angiotensin converting enzyme (ACE), partial cross-immunity from other respiratory corona viruses and possibly a better alveolar clearance in children [14], [15], [16], [17], [18]. 

As far as has been investigated, the most common transmission route to children is intra-family transmission from an adult family member to a child. The first comprehensive WHO report from China in February 2020 described that most clusters (78–85%) occurred in families [19]. In their review article published in March 2020, She et al. cite 9 publications (case reports and cluster studies) with intra-family transmission from adults to children [10]. At the beginning of April 2020, Viner et al. published a systematic review on the effects of school closings: based on 16 publications on SARS, including six on SARS-CoV-2, they did not find any reliable evidence of a relevant significance of school closings in reducing COVID-19 transmission, in contrast to the positive effect confirmed in many studies in influenza pandemics [20]. On June 11^th^ 2020, Rajmil [21] published a rapid scoping review regarding the importance of children in the transmission of SARS-CoV-2: 12 publications (case reports, case series and reviews) showed intra-family transmissions from adults to children, and two school-based publications [22], [23] did not provide any evidence of transmissions during regular school operations [21].

It should be noted, however, that in most countries extensive measures were taken to reduce transmission, including closing of children's community facilities. This reduced the likelihood of transmissions in child daycare centers and schools, but increased the probability of intra-family transmissions. Reports on studies on the spread of COVID-19 while schools were still open are available from only a few countries [21], [23], [24], [25].

Comparable evaluations for children are not yet known from Germany. Therefore, the data on mandatory reporting of SARS-CoV-2 in children in the city of Frankfurt am Main was evaluated with regard to the parameters:

- Comparison of age-related incidence and symptoms – children/adults
- Evaluation of the individual reporting data according to the possible path of infection of the children
  - Transfer within the family
  - Transfer within children’s community facilities (day care center, school, etc.)

The results are presented here, taking into account government measures to contain the corona pandemic, such as the temporary closure of schools and other children’s community facilities as well as the gradual reopening of those facilities. Together with previously published data, they should serve as a basis for discussing the resumption of normal school and daycare operations.

## Materials and methods

In the Frankfurt Health Department, all mandatory reporting data is documented and processed using the Survnet system [26]. For each case, the following data are entered: personal data (name, date of birth, age, gender, place of residence), disease characteristics (date of notification, date of onset of illness, symptoms), possible contact persons (family, others), and possibly care or work in medical facilities, children’s community facilities and schools, facilities for asylum seekers, or the food industry (according to the German Law of Infection prevention Law, Sections 23, 33, 36, and 42, respectively) [27].

In the first step, a query was made about all reports (reporting date, reporting week, age, activity in the facilities according to IfSG), and age-specific incidences were calculated.

In the next step, all reports from children 0 to 14 years of age were further checked in detail and evaluated according to symptoms, documented contact persons, possible path of infection, as well as attendance at kindergartens or schools according to § 33 IfSG [27] and/or refugee accommodations according to § 36 IfSG. Attending a facility was only noted as positive if it actually took place during the incubation period (not whether the child generally attended a daycare center or school). In order to obtain all information for each child, not only the drop-down fields provided in the program were examined, but also the “notes”.

## Results

**Figure 1:**
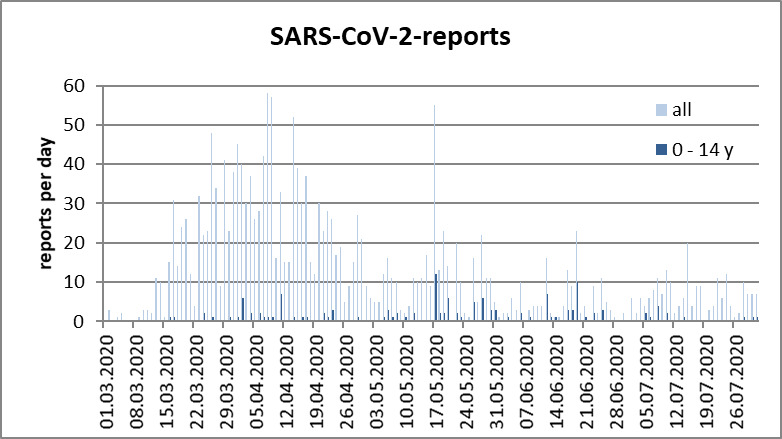
History of reports on Sars-CoV-2 infected people in Frankfurt am Main from March 1 to July 31, 2020. All 1,977 reports are compared with reports from 138 children aged 0–14 years.

From March 1 to July 31 2020, 1,977 people were reported with Coronavirus disease 2019 in Frankfurt am Main, including 138 children aged 0–14 years. Figure 1 (Fig. 1) exhibits the daily reports for the general population and children under 15 years of age. Because of large serial testing in various refugee accommodations, there were markedly high numbers of reports on May 17 as well as on June 12, 2020.

The SARS-CoV-2 incidence in the total population was 256/100,000, the age-related incidence in children was lower: 0–4 years 142/100,000, 5–9 years 132/100,000, and 10–14 years 178/100,000.

34 of the children lived in refugee accommodations in Frankfurt am Main, in which the residents were intensively tested for SARS-CoV-2 after the first individual reports in May and June 2020. 14 infected children were assigned to a transregional cluster originating a parish; the families in question had contact not only within their families but also during church services in May 2020. Here, too, extensive tests of the contact persons were carried out after the first known cases. Finally, 90 children neither lived in a refugee accommodation nor were they assigned to the cluster in the parish. They either only had contact at home or were also cared for in children’s community facilities.

Children reported symptoms much less frequently than adults; severe respiratory symptoms or the need for ventilation were neither observed nor noted. 62% of the children had no symptoms at all, compared to 19% of the adults; 5% of the children were admitted to hospital (adults: 24%), none died (adults: 3.8%) (Table 1 (Tab. 1)). 

When reviewing the files for possible transmission routes, children from the parish and the refugee accommodation were not taken into account, as no precise case history was possible in the context of the meetings or the close contacts in the accommodation with communal kitchens etc. Of the 90 cases neither living in a refugee accommodation nor assigned to the cluster in the parish, 4 (4.4%) infections had been acquired abroad. In 5 (5.5%) cases, transmission at the daycare center/school was possible/likely (two of them in facilities outside Frankfurt), and in two other cases, transmissions were reported between children from different households via visits or as overnight guests. 70 (77.7%) cases had been transmitted within the family from an adult family member to the child; in 4 (4.4%) of the cases, the transmission route could not be determined with certainty, as children and parents had been tested simultaneously, and in one case, the child was definitively tested positive before the adult household members, so that a transfer from child to adult is possible. Extraordinary test indications existed in 3 children: 1x before visiting the grandparents (risk group for COVID-19); 1x before a planned transplantation; 1x because of training in a sports club. In one case, the reason for the test could not be found out (Table 2 (Tab. 2)).

## Discussion

### Own data

Before discussing the data, the limitations should be mentioned. This is an evaluation of mandatory reporting data, not data of a standardized, prospective, scientific study.

1. The results depend on the availability of the tests, test strategy, occurrence of clusters in the municipality, and the type and scope of event-related screening examinations.
  In the first weeks of the pandemic, testing capacity was low, and the test strategy of the Robert Koch Institute initially focused on travelers returning from risk areas and their symptomatic contact persons. As of March 24, 2020, testing priority was given to symptomatic people from nursing homes – even without anamnestic COVID-19 contact – as well as staff. Children were to be examined to clarify outbreaks (family, school, etc.). These test strategies were implemented in Frankfurt am Main. For instance, in the first few weeks, returnees from risk areas were tested as a priority, and in the next few weeks, symptomatic people and/or contact persons of COVID-19 patients were tested (outbreak investigations). Only in the further course were children also tested after a possible contact in a children’s community facility, usually with an adult (teacher, caretaker, etc.) who tested positive. In May, after various reports from refugee shelters and at a parish, extensive screening examinations were carried out, regardless of any symptoms of the children or current close contact with a person who tested positive for COVID-19. It should be borne in mind that the more tests are performed, the more likely asymptomatic people will be tested positive, thus increasing the number of cases.
2. The results are also influenced by social conditions. If children’s playgrounds are closed and visits to child facilities are forbidden or restricted, the time spent in the family and thus the risk of infection within the family increases, while at the same time the risk of infection outside and in the community facilities decreases.
  In the State of Hesse, children’s facilities and schools were closed on March 16, 2020, with emergency care being offered for children of parents who work in the critical infrastructure. As of April 27, 2020, schools gradually reopened for the higher grades, obeying extensive preventive measures (small groups, distance 2 m, marked routes for in-school movement, lessons in person only every other day, with e-learning in between. As of May 18, all age groups, including the primary schools, returned to school and care facilities, adhering to strict hygiene requirements. Starting on June 22, regular operations during the COVID-19 pandemic took place with daily school lessons in person while still keeping to high hygiene standards; this was continued until the start of the school holidays on July 6, 2020.
3. It is not known how many families took advantage of the emergency care facility for their children, and how many children were actually cared for in a children’s community facility and potentially exposed in this context.
4. In mandatory reporting data, only the data of those who were tested positive or who became ill are available; the number of negative tests (or the total number of tests) is unknown, so that no positive rate can be determined. Likewise – as opposed to a prospective study – the data collected from the mandatorily reported cases, e.g., on symptoms, cannot be evaluated and presented in comparison with people who tested negative.
5. In the respective period, the data were not only collected by highly experienced employees of the health department, but – due to the abundance of the reports– also by non-specialist employees from other municipal departments and later by medical students as well as so-called “Corona scouts”. Thus, up to 60 people investigated the cases and entered the data in Survnet [26]. Despite instruction, it is possible that the usual quality in determination and documentation was not maintained
6. The time of sampling after suspected contact or after infection and the type of sampling (brushing technique) as well as further processing in the laboratory can influence the results. In the case of reporting data in which many different actors are involved, quality assurance as in standardized prospective studies is not possible. In general, testing too early after a potential infection leads to a false negative result.
7. On the other hand, if the prevalence in the population is low, a low positive predictive value and therefore false positive results may be expected – even in tests with high sensitivity and specifity [28], [29].
8. “Genetic fingerprinting” to check whether the virus detected in adults and their children is identical was not carried out. Both adults and children could have been infected by various index cases and an epidemiological connection could not possibly be established.

Despite the above limitations, when evaluating registration data, all people testing positive and living in the municipality are mandatorily reported, regardless of the test location and reason. Thus, local and regional incidences can be calculated – for the population in total as well as for different age groups. In addition, with regard to SARS-CoV-2, it can be assumed that all laboratories have reported completely and therefore underreporting of positive test cases is unlikely.

Hence, the following can be stated here:

1. Only a few children tested positive in relation to the general population who tested positive as well as to the calculated incidences

Within Germany, comparable test capacities and strategies as well as largely identical social conditions (protective measures, shut down of children’s care facilities) can be assumed. The SARS-CoV-2 incidence for the population in Frankfurt is (as of July 31, 2020) 286/100,000 inhabitants. This compares well with the total incidence in Germany, which is 256/100,000 inhabitants [30].

The age-specific incidences of children in Frankfurt (as of July 31, 2020) are significantly lower: 0–4 years 142/100,000, 5–9 years 132/100,000, and 10–14 years 178/100,000. In Germany as a whole, the age-specific incidences are even lower: 0–4 years 92/100,000, 5–9 years 86/100,000, and 10–14 years 115/100,000 (as of August 1, 2020). A possible explanation for the higher incidences among Frankfurt children compared to nationwide could be that extensive test series were carried out in Frankfurt due to COVID-19 infections in a parish as well as in refugee accommodations, in which a comparatively large number of children tested positive. Taking into account only those children without any relation to the parish or to refugee accommodations, the age-specific incidences coincide with those of Germany overall.

Lower age-specific incidences in children than in adults were also reported from other countries, in spite of different test strategies and social conditions, such as school closings, population density, etc. It is particularly interesting to compare two Scandinavian countries with an overall low population density and different mean of combatting the COVID-19 pandemic: Finland and Sweden [25]. While kindergartens and elementary schools remained open in Sweden, schools in Finland were closed from March 18, 2020 to May 13, 2020, with emergency daycare being offered for children in grades 1–3 whose parents work in the critical infrastructure. Until June 14, 2020, in Finland the incidence among 0–5 year olds was 36/100,000, and among 6–15 year olds it was 42/100,000. Astonishingly, the corresponding incidences were lower among children in Sweden: 0–5 yrs: 16/100,000, 6–15 yrs: 30/100,000. The authors conclude that closure or not of schools in Finland or Sweden had no measurable effect on the number of laboratory-confirmed SARS-CoV-2 infections in school-aged children.

Thus, the low SARS-CoV-2 incidence in children is “stable” even in different countries with very different pandemic hygiene procedures.

1. Compared to the adults, the children with laboratory-confirmed SARS-CoV-2 infections were often asymptomatic or exhibited low symptomatology

Compared with adults (≥20 y), children under 15 years of age in Frankfurt were much more likely to have no symptoms (62% vs. 19%), they were admitted to hospital less often (5% vs. 25%) and did not report severe respiratory distress symptoms or require ventilation. No child died.

Although these results are heavily influenced by the test strategy (e.g., whether only symptomatic children are examined on an individual basis or whether asymptomatic children are also tested in the context of larger environmental studies (e.g., refugee accommodation)), the low rates of laboratory-confirmed SARS-CoV-2 infections in children also agree with numerous reports from different countries with different test strategies [3], [4], [5], [6], [7], [9], [10], [11].

1. The majority of the children were infected by an adult family member within the family environment, not while attending children’s community facilities.

A detailed review of the files showed that 78% of the children were infected by adult household members within the family; in one family only, the virus had apparently been passed on from one child to an adult family member. In 4 families, the children and adults tested positive on the same day and the exact transmission route could not be determined. In 5 cases, transmissions in a children’s community facility were established as possible, including 2 cases who had been infected in another federal state before moving to Frankfurt a few days later.

This data is also in agreement with early reviews [10], [11] as well as another paper published at the beginning of August, in which the data of hospitalized children under 16 years of age in Geneva was reported [31]: There, in 31 of 39 (79%) cases, an adult household member tested positive before the child became positive; in 3 (8%) cases, children tested positive before an adult household contact.

1. There are no hints that the number of infected and positive children increased significantly after partly reopening children’s community facilities.

Taking into account the special situation in the parish and the fact that the children tested in refugee accommodations in May had not yet returned to school at that time, no increase in the number of SARS-CoV-2-positive cases in connection with the gradual reopening of schools (under observance of strict hygiene measures) was recognizable.

In Finland, there was no increase in SARS-CoV-2 infections in children after reopening of the schools in mid-May [25]. 

Our data thus confirms many studies, including a current narrative review on the published evidence about transmission of SARS-CoV-2 by children, encompassing household cluster studies, school outbreak investigations, seroprevalence, and clinical laboratory as well as time series studies [32]: 

Children are not the main spreaders – neither in the family nor in the school setting. 

### International data on COVID-19 and attending school

Various studies with different methods from different countries are available on schools and transmissions in childcare centers and schools.

#### Studies related to schools

##### New South Wales, Australia

The schools remained open the entire time, but from March 23, 2020 onward, parents were encouraged by the government to keep their children at home for online learning. Between March 5 and April 4, 18 COVID-19 cases (9 students and 9 staff) were identified in 15 schools. (Schools with index case: one school each on March 5, 9, 20 and April 1, 2, 3, 2020, and two schools each on March 6, 23, 24 and 25). Contact persons were identified by the public health service. 863 contact persons, including 735 schoolchildren and 128 employees with close contact, defined as at least 15 minutes of face-to-face contact, accepted the offer of intensive surveillance. This comprised a symptom questionnaire, a throat swab for SARS-CoV-2 5 to 10 days after contact with the index case and an antibody test after 4 weeks. Half of the index cases in more than half of the schools (4 out of 5 primary schools and 4 out of 10 secondary schools) were school staff. The investigation of the contact persons revealed no evidence of children infecting teachers. Although two students identified as secondary cases, the authors concluded that it was most likely, but not certain, that they were infected by transmission in the school environment [23].

##### Ireland

In Ireland, schools were closed from March 12, 2020. A review of the registration data revealed 6 cases (3 children and 3 adults) who had previously attended their schools although already being contagious. Two of these cases had acquired SARS-CoV-2 on travels, one of them had subsequently infected 2 household members and one case each had been infected in a recreational context or at work (not school). Five cases had symptoms of COVID-19 disease, one child (from the family cluster) was asymptomatic. A total of 1,155 contact cases were identified for these 6 cases, including 1,025 school contacts. The school contacts included lessons in the classroom, sports lessons, music lessons, and choir practice for a religious ceremony, in which various schools participated. The contact persons were informed by the public health service and asked to report any symptoms that might develop. Symptomatic contacts were tested regardless of the intensity of the contact or the severity of the symptoms. While there were two transmissions between adults in the context of out-of-school contacts, not a single one of the 1,025 school contact persons tested positive within the next few weeks. In the following time, there were also no cases that could be related to school attendance at that time [22].

##### Israel

Schools in Israel [33] were closed on March 13, 2020 and reopened for certain classes and small groups on May 3, 2020. From May 17, 2020 onward, all schools were fully opened again – with requirements for daily health reports, hygiene, face masks, social distancing, and minimal interaction between classes. On May 26 and 27, 2020, two students from one school tested positive. Since they had still attended school during their infectious period, all schools in Jerusalem were closed again and a mass test of students and teachers in the respective school was performed with 151 employees and 1,161 students taking part: 153 (13.2%) students and 25 (16.6%) employees were confirmed as SARS-CoV-2 positive. In addition, 87 other close contacts outside of school tested positive. The positive rate was higher in the 7^th^ to 9^th^ grades than in the 10^th^ to 12^th^ grades. The authors report that distancing had not been possible in full classes (35–38 students on 39–49 m^2^), and that because of a strong heat wave, face masks had not been worn in many cases. Looking at the weekly reporting data in the city of Jerusalem, in the 22^nd^ reporting week, there was an enormous increase in reports for children aged 10–19 to around 160 cases and a further 75 cases in the following week. These numbers are not much higher than the number of positives from this affected school. Therefore, it can be concluded that there was a special problem in this school and that other schools in the city were not or less affected. Unfortunately, the paper does not state when symptoms started in children or teachers, so that transmission from children to teachers and vice versa is likely. The media reported that the outbreak in this school was apparently due to a teacher as “superspreader” – but what this assumption is based on remains unclear. 

#### Population studies

##### Iceland

In Iceland, childcare facilities and elementary schools remained open, but general “interpersonal distancing” was recommended from March 16. Large gatherings as well as visits to hospitals and nursing homes were banned. Universities and colleges were closed. Three population studies were carried out using throat swabs and PCR testing: a) a targeted examination of 9,199 symptomatic persons and contact persons from January 31 to March 31; b) an open population screening from March 13 to April 1 with 10,797 people tested; and c) a randomized population study encompassing 2,283 people from April 1 to 4. In the targeted examination, 13.3% (6.7% of the children under 10 years of age) tested positive, and in the population studies 0.8% and 0.6% had a laboratory confirmed SARS-CoV-2 infection (not a single child under 10 years of age). Hence, there was no indication that children were infected by continuing operation of the children's facilities and primary schools [24].

##### Italy

In Vo, a small town with 3,275 inhabitants in northern Italy, two population surveys (throat swabs and PCR testing for SARS-CoV-2) were carried out after one patient in the town died of COVID-19 on February 21, 2020. Three days after this death, a lockdown of the whole municipality was imposed for 14 days. In the first survey from February 22–29, 2020, 86% of the population was examined for SARS-CoV-2; in the second survey at the end of the lockdown on March 7, 2020, 72% of the population took part, including 217 and 157 children under 10 years, i.e., 96% and 68% of this age group, respectively. The prevalence of those who tested positive was 2.6% in the first survey and 1.2% in the second. In neither of the surveys did children under the age of 10 years test positive, even though they had attended schools and kindergartens until the lockdown, and some of them also lived in families with adults who tested positive [34].

##### Finland

In Finland, schools were closed from March 18 to May 13, 2020, except for emergency access for children whose parents worked in critical infrastructure. Sweden, on the other hand, let elementary schools and kindergartens remain open throughout the whole period. In Finland, the number of mandatorily reported cases was no lower during the school closure than after reopening. The age-specific incidences (as of June 14, 2020) were significantly lower in Sweden than in Finland. The authors concluded that the school closings had no measurable effect on the number of reported COVID-19 cases in children and that children are neither a major risk group for COVID-19 nor do they play a significant role in transmission [25].

##### Situation in Germany

Given the experience with influenza, where children often accelerated the epidemic and school closings could reduce the spread, schools and childcare facilities were closed in many countries, including Germany. Through emergency care for children of parents working in the critical infrastructure – including the medical and nursing fields – the risk of limited medical care and thus an increasing risk of mortality in the population due to a lack of staff due to private care for their children [35] was counteracted. In international literature, early attention was given to the negative effects on the general and mental health of children, health and social effects of school closings [20], [36], [37]. Considering the findings that children are not super-spreaders, are usually infected by adults and not the other way around when the virus is transmitted within families, and that there is a lack of evidence for school-based transmissions, attention was drawn to the right of children to education, and school closings were re-evaluated or their early reopening was demanded [38], [39].

Pediatric societies in Germany published the first statements on children and COVID-19 as early as March. As early as April, taking the needs and rights of children and adolescents into consideration, pediatric societies and networks [40], [41], [42] called for a rapid normalization of the situation for children. On April 20, 2020, the German Academy for Child and Adolescent Medicine (DAKJ) recommended school reopening as soon as possible [43]. In a further statement dated May 4, 2020, with reference to the publications of various pediatric specialist societies (neuropediatrics, pediatric cardiology, pediatric immunology, child and adolescent rheumatology, pediatric diabetology) it was emphasized that “potentially exaggerated protection intentions can do more harm than good” [44].

At the end of April, a paper was published on the viral load in the throats of children compared to the elderly [45]: The authors concluded from their data that children do not have lower viral loads than adults and therefore considered SARS-CoV-2 in children being as likely to be transferred as in adults. When this paper and in particular the statistical methods used were criticized by various statisticians [46], [47], the authors revised their publication and included further data. According to the authors, the lower viral load in children (“a credible but small difference”) found with a newer analysis method, and the less frequent exceeding of a certain viral load associated with the presence of infectious virus particles, were more likely due to different modes of handling of the test instruments than to real differences. The conclusion of the authors was again: “There is little evidence from the present study to support suggestions that children may not be as infectious as adults” [48].

However, this conclusion is not permissible [38]: On the one hand, children have less breath volume and weaker coughing than adults. On the other hand, all epidemiological data – despite their methodological limitations – provide no indication of a higher transmission rate in children than in adults.

While the Jones study received broad media attention, a joint statement by the German Society for Hospital Hygiene (DGKH), the Association of Pediatricians (BVKJ), the German Society for Pediatric Infectious Diseases (DGPI), the German Academy for Children's and Adolescent medicine (DAKJ) as well as the Society for Hygiene, Environmental Medicine and Preventive Medicine (GHUP) [49] received little to none. In this statement, with reference to the current data basis, the professional societies recommended the following, among other things:

„Taking into account regional infection rates and available resources, daycare centers, kindergartens and elementary schools should promptly be reopened. For children, this should be possible without excessive restrictions, such as clustering into very small groups, implementation of barrier precautions, maintaining appropriate distance from others or wearing masks. A factor more decisive than individual group size is the sustaining of constancy of respective group and the avoidance of intermixing.

Children can be taught basic rules of hygiene such as handwashing and careful hygiene behavior when coming into contact with others during mealtimes and/or when using sanitary facilities. This can be done in a playful and age-appropriate way. Based upon current knowledge, the implementation of such instruction, together with the mandatory equipment of all school bathrooms and handwashing sites with sufficient soap dispensers and paper towels would have considerable, positive, long-term effects on the spread of many different contagious pathogens in these facilities [...]” [49].

Furthermore, they emphasized that “in contrast to homes for the elderly, community facilities for children and adolescents do not represent a high-risk environment per se”. In addition, the detection of individual infections in children or schoolchildren should not automatically lead to the closure of the entire daycare or school. Rather, a detailed analysis of the infection chains is required for balanced infection management [49]. Similar recommendations have been published in Norway, which reopened daycare centers after a lockdown in March 2020 on April 20, 2020, elementary schools on April 27, 2020 and schools for higher grades on May 11 [50].

Simultaneous with the end of the school holidays, the German Academy for Pediatrics published another paper, with differentiates measures to facilitate school openings [51]. 

These statements by scientific societies do not stand alone. In their thesis paper of April 5, 2020, Schrappe et al. already referred to the importance of target group-specific prevention strategies (elderly and people with comorbidities), as well as the importance of nosocomial contact and the occurrence of clusters. They referred to social inequality and psychosocial implications, especially in children [52]. In their Statement 2.0 of May 3, 2020, they wrote: “Hardly any other topic has been discussed as controversially as the role of children in the infection process (opening of day care centers, compulsory schooling, etc.)” Following available epidemiological data they conclude: “From a scientific point of view, there is no justifiable reason to prevent the reopening of childcare and educational facilities for children” [53].

It is particularly important that the well-known hygiene problems in schools, e.g., insufficient ventilation, insufficient cleaning, hygiene problems in restrooms (lack of equipment, vandalism) [54], [55], [56], receive greater attention and finally be remedied. In the summer of 2009, when the H1N1 pandemic (“swine flu”) was already virulent in the southern hemisphere, the Frankfurt am Main health department informed all schools and children’s community facilities about the necessity for good hygiene facilities, especially for hand hygiene. In September, 455 washbasins in 62 schools were checked and many problems were found. During the follow-up checks in November 2009, shortly before the swine flu reached Germany, all necessary improvements had been completed [57].

Similarly, the Frankfurt am Main health department carried out hygiene inspections in 110 schools in Frankfurt from mid-May to the end of June 2020: more than 95% of the schools had adjusted their hygiene plan, and the sanitary units were well equipped; the schools exhibited a good hygienic situation. In more than 85% of the schools, the required distance between the school desks was kept and it became obligatory for children to cover their face and nose outside the classroom [58]. 

However, there is also a need for politicians to better inform and communicate with the population. Instead of communicating worst-case scenarios only and threatening another lockdown in order to achieve “the desired shock effects”, and thus address the “primal fears” and feelings of guilt of the population (BMI quoted from Schrappe [53]), politicians should switch to a constructive, positive presentation focused on the competences of citizens.

After the publication of its concept for strategies for mitigation and protection of vulnerable population groups from the COVID-19 epidemic, taking into account the proportionality of March 23, 2020 (DGKH March 23, 3030), the DGKH (German Society for Hospital Hygiene) made a further assessment of the situation on March 30, 2020: 

“The differentiation of the population groups according to threat from severe infection, intensive care, ventilation and death and not according to risk from mere infection with generally little or no symptoms is of the greatest importance. Increasing numbers of infections in general are secondary and only important insofar as the risk of transmission must be controlled. Infections among the non-threatened age and non-risk groups could even, through the development of a natural immunity (focal immunity) due to an infection, help slow the spread of the pandemic and bring it under control, as long as active immunization through the availability of a vaccine does not exist” [59].

Given that a vaccination against SARS-CoV-2 will be unavailable for many more months, and considering the data reported above, instead of continuously following the concept of containment with ongoing mandatory school closures when a case has been detected, there should be a comprehensive, open discussion in society at large on “protection” of the risk-groups and on “mitigation” – thus following the German Concept of the Robert Koch Institute for Pandemic situations [60].

## Notes

### Competing interests

The authors declare that they have no competing interests.

## Figures and Tables

**Table 1:**
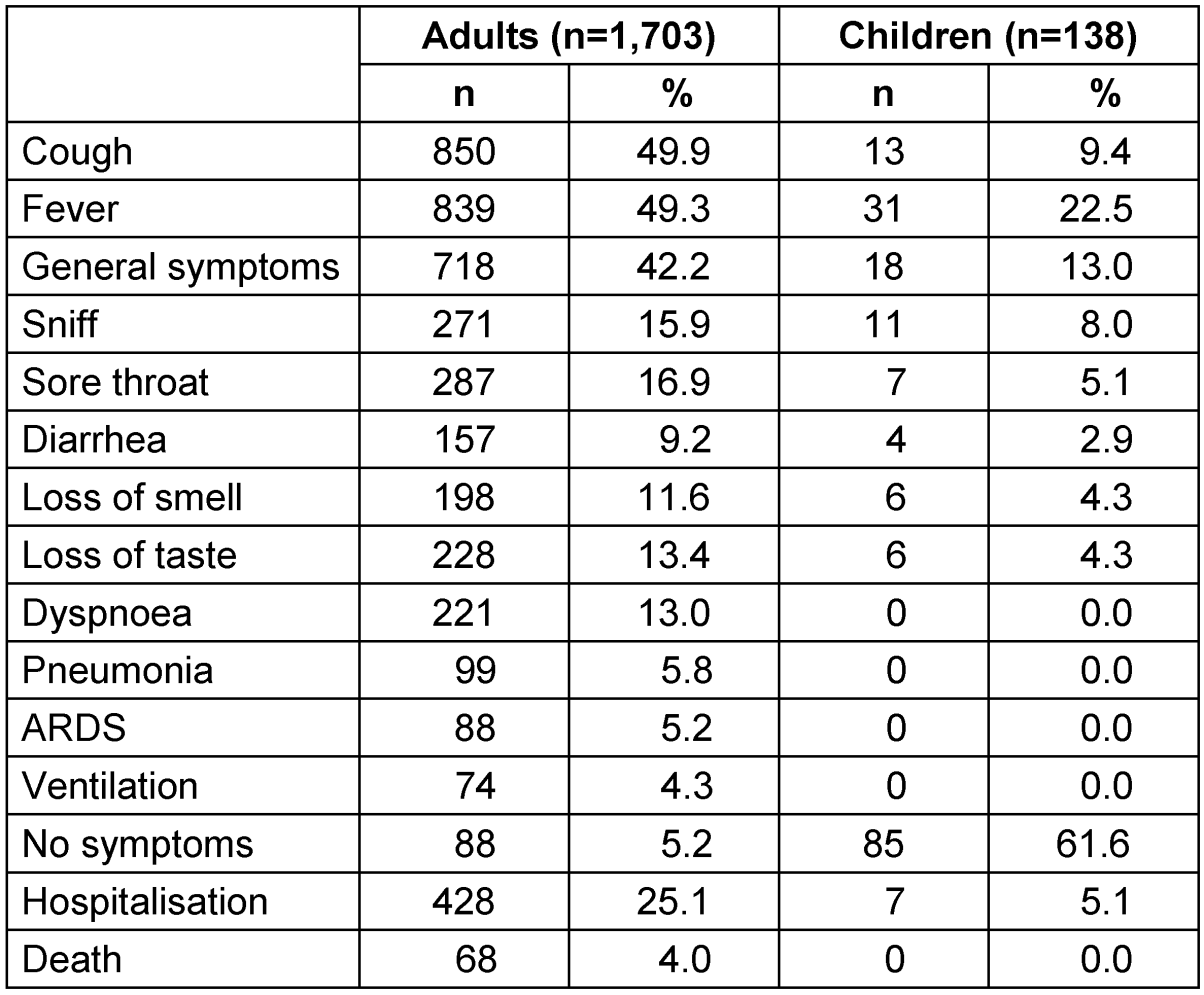
Symptoms in children and adults who test positive for Sars-CoV-2

**Table 2:**
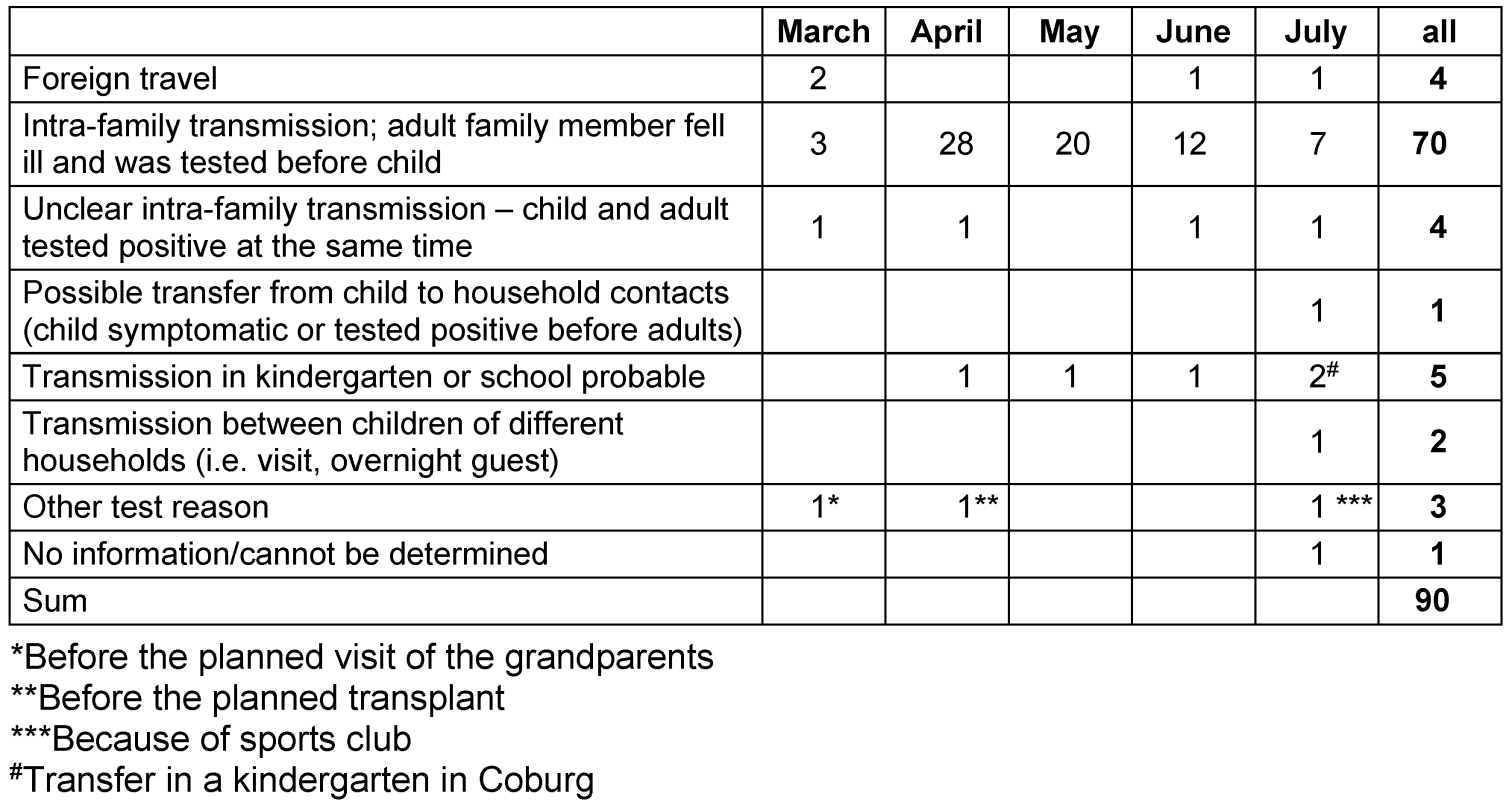
Probable transmission routes of 90 children who tested positive for SARS-CoV-2 (excluding children from the parish cluster and refugee accommodations)
